# A natural seaweed derived mineral supplement (Aquamin F) for knee osteoarthritis: A randomised, placebo controlled pilot study

**DOI:** 10.1186/1475-2891-8-7

**Published:** 2009-02-02

**Authors:** Joy L Frestedt, Michael A Kuskowski, John L Zenk

**Affiliations:** 1 Frestedt Incorporated, 2708 Vernon Ave S, St. Louis Park, MN 55416, USA; 2 Department of Psychiatry, University of Minnesota Medical School, Minneapolis, MN, USA; 3 Humanetics Corporation, 10400 Viking Drive, Suite 100, Eden Prairie, MN 55344, USA

## Abstract

**Background:**

Osteoarthritis (OA) is a slowly destructive process that may be influenced by a nutritional mineral balance in the body.

**Methods:**

This small, double blind, placebo controlled pilot study investigated the impact of treatment with a natural multi-mineral supplement from seaweed (Aquamin) on 6 minute walking distance (6 MWD), range of motion (ROM), and pain and joint mobility measured by the Western Ontario and McMaster Universities (WOMAC) Osteoarthritis Index in subjects with moderate to severe OA of the knee during gradual withdrawal of non-steroidal anti-inflammatory drugs (NSAIDs) that were being used daily for pain management. Subjects (n = 29) with moderate to severe OA of the knee were randomised to receive either Aquamin (2400 mg/d) or Placebo for up to 12 weeks.

**Results:**

Of the 29 subjects initially randomized, only 22 subjects proceeded to treatment due to 7 subjects not meeting study selection criteria at baseline. Fourteen subjects completed the study and an ITT analysis (n = 22) of the data showed no significant differences in WOMAC scores however, the data did reveal significant improvements in passive and active extension ROM (0.83° ± 1.54 vs. -1.54° ± 2.43; difference, 5.2° ± 2.2, p = 0.028) and 6 MWD (150 ± 48 ft vs. 12.5 ± 31.5 ft; difference, 136 ± 57 ft, p = 0.03) in the Aquamin group compared to the placebo group; respectively, following a 50% reduction in NSAID use. The treatments were well tolerated and the adverse event profiles were not significantly different between the groups.

**Conclusion:**

This small preliminary study suggests Aquamin may increase range of motion and walking distances in subjects with OA of the knee and may allow partial withdrawal of NSAIDs over 12 weeks of treatment. Additional research is needed to confirm these preliminary observations.

**Trial registration:**

NCT00755482

## Background

Osteoarthritis (OA), also called degenerative joint disease, is a slow destructive process of the joint. Although the exact biochemical cause of OA remains unknown, the process usually begins when the joint structures are abnormal or the stress placed on the joint surfaces is unusually high. Much of the disease progression is due to mechanical stress on the joint and over time the excessive stress can induce metabolic and structural changes in the cartilage, bone and articular surfaces. In addition, bone and joint abnormalities have been documented in developing countries and may be associated with nutritional and developmental problems. OA is expected to affect over 59 million people in the United States alone by the year 2020 [1]. In industrialised countries, primarily older people suffer from this disease which is aggravated by increasing weight with age, increasingly sedentary lifestyles and the lack of adequate exercise regimes.

Non-steroidal anti-inflammatory drugs (NSAIDs) are frequently employed to provide relief from symptoms of OA but NSAID use is sometimes accompanied by unwelcome side-effects including gastrointestinal distress, ulcer formation and cardiovascular problems [2,3]. As a result, glucosamine and other new anti-inflammatory compounds showing reduced adverse effects are being explored as possible treatments for OA [4-6]. Growing evidence suggests that several minerals play an important role in joint health. Naturally occurring minerals such as magnesium, copper, manganese, selenium and zinc have shown anti-inflammatory effects in both animal and human studies. In a rat model of osteoarthritis, a deficiency of dietary magnesium increased cartilage damage [7]. Trace minerals such as boron and manganese have been shown to reduce the symptoms and may slow the pathogenesis of OA [8].

Calcium supplementation has been shown to improve bone mineral density and recent reports suggest that calcium may also play a role in ameliorating the symptoms of OA. For example, the administration of 1 gram of calcium ascorbate daily for a period of 14 days to 133 patients with OA of the knee and/or hip resulted in a significant reduction in pain compared to placebo [9].

A calcium and magnesium-rich seaweed-derived multi-mineral supplement, Aquamin, was investigated in two similarly designed trials conducted in the same clinic at the same time with two independent groups of subjects. One trial was previously published showing improved symptoms of OA including improved Western Ontario and McMaster Universities (WOMAC) Osteoarthritis Index [10] scores and walking distances in subjects consuming Aquamin compared to placebo [11]. Aquamin, is a multi-mineral supplement, derived from the red algae Lithothamnion corallioides which is rich in calcium and magnesium and a variety of trace minerals including manganese, selenium and zinc (Table 1). This preliminary study was designed to investigate the potential for Aquamin to allow reduced NSAID usage over three months in subjects with moderate to severe OA of the knee.

**Table 1:** Typical mineral composition of Aquamin*

Mineral	Dry salt weight
Calcium	34%
Magnesium	2.4%
Phosphorous	0.08%
Potassium	0.7%
Sulphur	0.67%
Iron	915 ppm
Boron	16.5 ppm
Sodium	0.225%
Manganese	125 ppm
Cobalt	0.1 ppm
Copper	10 ppm
Zinc	26.1 ppm
Selenium	1 ppm

* Aquamin contains a wide spectrum of trace minerals assimilated from sea water. Aquamin is a natural ingredient and trace mineral levels may vary over time.

## Methods

### Study design

This small preliminary randomised, double blind, placebo controlled parallel group study investigated the effect of Aquamin on NSAID dose reduction in subjects with OA of the knee. We used anecdotal information along with our experience from a previous study with a similar endpoint to estimate the number of subjects that might be needed for this small pilot study [12].

### Ethics

This study was reviewed by Quorum Review Inc, a central IRB (Seattle, WA), performed in compliance with all the applicable regulations and guidelines (e.g. Good Clinical Practice Guidelines, the Declaration of Helsinki, 21CFR50-Protection of Human Subjects and 21CRF56-Institutional Review Boards) and was monitored and audited according to Minnesota Applied Research Center Standard Operation Procedures (SOPs). Subject data was kept confidential and study records were stored in a locked and secure storage area.

### Study center and subject selection

This study was conducted at the Minnesota Applied Research Center (Edina, MN). Subjects of either gender were included if they voluntarily gave informed consent, were ambulatory, 35–75 years old, with normal digestion and absorption and were diagnosed with symptomatic moderate to severe OA of the knee according to their previous medical history and the modified clinical criteria of the American College of Rheumatology [13]. All subjects were required to have a WOMAC total scores ≤ 75 at entry and were currently taking NSAIDs daily for pain management. The target knee was identified by physical examination as the most severely affected knee for each subject and the cut off point for the WOMAC score was enforced as a means of standardizing the extent of pain and immobility in the small number of subjects recruited for this trial. A waiver to the selection criteria was made for one subject who had a WOMAC score of 76 and was enrolled in the trial.

Subjects were excluded from this trial if they had rheumatoid arthritis, gout, pseudogout, Paget's disease, seizure disorder, insulin dependent diabetes mellitus, uncontrolled hypertension, unstable cardiovascular disease, active hepatic or renal disease, active cancer, HIV infection; if they required prescription drugs to control pain; if they had other clinical trial or experimental treatments in the past 3 months; if they were pregnant, lactating or at risk of becoming pregnant; or if they had intramuscular or systemic corticosteroid administration within 1 month, intra-articular corticosteroid injection within 2 months or intra-articular hyaluronic acid injection within 4 months prior to study enrollment.

### Study visits and treatments

The study was conducted over 14 weeks with a total of 6 visits to the study centre at -2 (screening), 0 (baseline), 2, 4, 8 and 12 weeks on treatment. During the initial two-week screening period (-2 to 0 weeks), subjects were asked to discontinue any prescription, over-the-counter or alternative therapy treatments for pain management but were allowed to use their preferred NSAID for pain management. In order to establish a standardised calcium intake across all treatments, subjects were asked not to change their usual dietary patterns during this trial except to consume a diet with approximately 600 mg calcium per day (e.g. two dairy servings) which was estimated to be 40–60% of their Recommended Daily Allowance (RDA) for calcium (depending on their age). Subjects were also asked to maintain the same amount of exercise throughout the study even if their joint pain was reduced. Qualified subjects were randomised (1:1) to receive either placebo or Aquamin and the trial activities remained double blinded throughout the trial to avoid bias. The subjects were blindly allocated to one of two treatment groups, according to a randomisation table, in the order of their enrollment in the study. The computer generated randomisation table was created by an independent statistician and the investigators were not aware of the group code assignments. A third party was responsible for labelling the test article and placebo according to the subject number and the randomisation table and the group code assignments.

At the baseline visit, all subjects received pre-packaged two-piece hard shell capsules containing either Aquamin (267 mg Aquamin + 167 mg maltodextrin) (34% calcium, 88.1 mg) or placebo (434 mg maltodextran). The bottles (and the capsules inside) appeared identical and subjects were instructed to consume three capsules, with a glass of water, three times per day. After taking either Aquamin or placebo for 2 weeks (0–2 weeks), subjects were asked to reduce NSAID use by 50% for a period of 2 weeks (2–4 weeks) and to eliminate NSAID use completely after 4 weeks and for the remainder of the study (4–12 weeks). Pain was managed with acetaminophen (325 mg per tablet with 1–2 tablets to be taken every 4–6 hours as needed for pain).

### Study measurements

Vital signs, adverse events and the amounts of acetaminophen used for pain were assessed at each visit. Laboratory tests were performed and subjects completed WOMAC questionnaires as well as 6-minute walking distance (6 MWD) and range of motion (ROM) tests. Subject compliance was assessed at each visit by pill count, interview, and review of the medication diary. The WOMAC Osteoarthritis Index is a validated questionnaire including scores for pain, stiffness and activity as well as a composite (total) score. The 6 MWD was conducted by marking off a 100-foot distance in an interior hallway and asking subjects to walk as far as they could as quickly as possible over 6 minutes. The total distance was measured and recorded. ROM tests for the effected knee were measured using a goniometer.

### Statistical analyses

An independent statistician analysed the data using t-tests (for single between-group comparisons), ANCOVA (for between-group comparisons at specific timepoints, using baseline score as a covariate) and a mixed linear regression model on repeated measures data (for between-group comparisons across all timepoints) to analyze data for an Intent to Treat Group (including all subjects enrolled and treated in this trial with values imputed for their Last Observation Carried Forward (LOCF) for any subjects who did not complete the trial) and a Completer's Group (including only data from subjects who completed the trial per protocol).

## Results

### Subject enrollment and disposition

This study was initially designed to enroll 50 volunteers; however, recruitment was terminated after 29 subjects were screened due to a lack of volunteers. The subjects expressed a fear that they would have OA pain symptoms following the complete discontinuation of NSAIDs as required for the study. Of the initial 29 subjects enrolled, 13 were assigned to the Aquamin group, 15 were assigned to the Placebo group, and one was not randomized to a group assignment. Seven (7) of the 29 subjects did not to fit the trial protocol requirements at their baseline visit and were discontinued from the study. Of these seven subjects, 5 were allocated to the Aquamin arm, 1 was allocated to the placebo arm and one was not yet randomized to a group assignment. Therefore, 22 of the initial 29 subjects went on to the treatment phase of the trial, with 8 subjects allocated to the Aquamin arm and 14 subjects allocated to the placebo arm.

**Figure 1:**
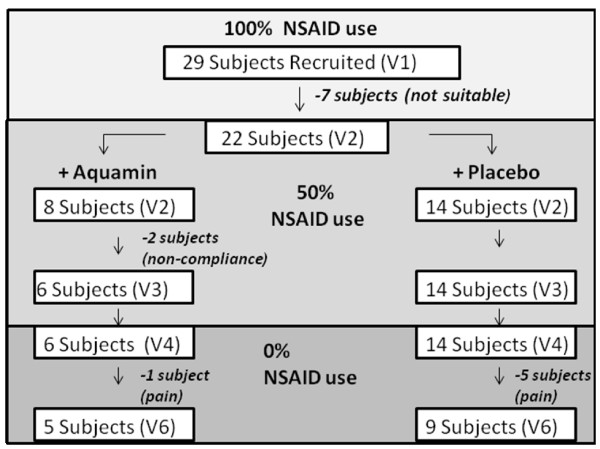
**Trial flow chart**.

A total of 14 (64%) of the 22 treated subjects completed the trial with treatment for the expected 12 weeks (84 days) (Table 2). Eight (36%) subjects withdrew from the trial including five subjects from the placebo arm and three from the Aquamin arm. Two subjects withdrew from the Aquamin arm at days 1 and 10 of treatment (prior to any reduction in NSAID usage) because they were unable to maintain adequate compliance with one or more aspects of the protocol and one subject withdrew from the Aquamin arm on day 65 due to increasing knee pain. In contrast, five subjects withdrew from the placebo arm due to increasing knee pain in the latter stages of the trial (41 +/- 14 days [mean +/- SD] – at 28, 32, 39, 41, and 64 days) when NSAID use had been completely eliminated. Although considerably more subjects withdrew due to increasing pain levels in the placebo group (n = 5) than the Aquamin group (n = 1) this was not statistically significant (Figure 1).

**Table 2:** Subject disposition

Trial ID	Placebo	Aquamin F	Total
Subjects Treated	14	8	22
Subjects Completed	9	5	14
Number Withdrawn	5	3	8
Withdrawn – Days in Trial *	41 (14)	25 (35)	35 (23)

*Values are given as the mean (standard deviation)

### Baseline characteristics

No significant differences were seen between the groups for any of the measured variables at baseline (0 weeks/V2; Table 3). Of the 22 subjects receiving treatment in this trial, seven females and one male received Aquamin (n = 8) whereas eight females and six males received placebo (n = 14). The mean age was 62.5 vs. 62.9 years and the mean weight was 91 vs. 94 kilograms for the Aquamin versus placebo groups respectively.

**Table 3:** Baseline demographics

Measurement	Placebo N = 14	Aquamin N = 8	P values*
Gender (F/M)	8/6	7/1	0.141
Age	62.9 (11.4)**	62.5 (5.3)	0.934
Height	68.3 (3.8)	65.5 (3.7)	0.144
Weight	94 (24)	91 (23)	0.753
Systolic Blood Pressure	142 (22)	133 (23)	0.394
Diastolic Blood Pressure	83 (13)	78 (12)	0.414
Pulse	69 (17)	78 (13)	0.183
Temperature	97.9 (1.0)	97.9 (0.4)	0.857
WOMAC – pain	60 (15)	54 (17)	0.428
WOMAC – stiffness	54 (21)	45 (15)	0.294
WOMAC – activity	57 (18)	49 (19)	0.327
WOMAC – composite	58 (16)	50 (18)	0.318
ROM – PF	76 (6)	73 (6)	0.661
ROM – AF	78 (14)	73 (16)	0.471
ROM – PE	170 (6)	172 (6)	0.497
ROM – AE	170 (6)	172 (6)	0.497
6 MWD	1289 (281)	1318 (267)	0.819

*P value based on t-test with equal variance assumed (except gender which was analysed with chi square analysis). **except for gender, values are given as the Mean (standard deviation)

### Test article and rescue medications

No significant differences were found between the two groups for test article or rescue medication consumption at any single timepoint or over time in this trial. Although not statistically significant, the use of pain meds increased over time from V4 to V5 to V6 for both groups indicating parallel courses for both groups over the time period of this trial.

### WOMAC

No significant differences were found between the two groups for WOMAC pain, stiffness, activity or composite scores (Table 4).

**Table 4:** Changes in study endpoints from baseline to visit 4

Measurement	Placebo** N = 14	Aquamin** N = 8	Group differences***	P values*
WOMAC – pain	5.38(2.80)	10.83(8.30)	3.5(7.1)	0.63
WOMAC – stiffness	4.81(4.37)	10.42(12.67)	1.9(8.8)	0.83
WOMAC – activity	6.54(2.16)	14.72(9.04)	5.5(6.8)	0.43
WOMAC – composite	6.17(1.96)	13.55(8.98)	4.9(6.7)	0.47
ROM – PF	3.84(2.48)	6.67(3.57)	2.7(4.3)	0.54
ROM – AF	1.92(2.63)	8.33(4.22)	6.0(4.8)	0.23
ROM – PE	-1.54(2.43)	0.83(1.54)	5.2(2.2)	0.028
ROM – AE	-1.54(2.43)	0.83(1.54)	5.2(2.2)	0.028
6 MWD	12.5(31.5)	150(48)	136(57)	0.03

* P value based on ANCOVA group effect. ** mean change (SE). ***mean group difference in change (SE) based on ANCOVA estimated marginal means

### ROM

**Figure 2:**
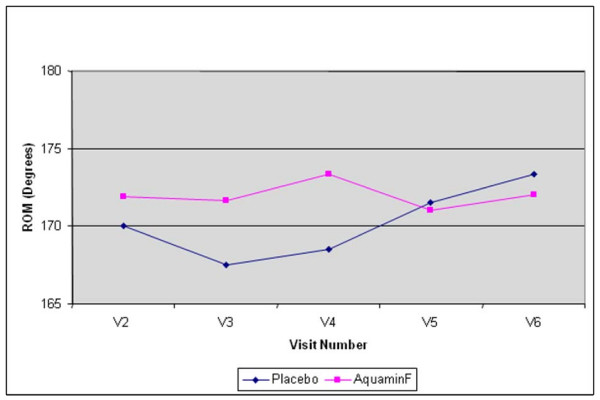
**Active and passive ROM over time**. Change (Degrees) in ROM over 12 weeks of treatment.

No differences were found between the two groups when ROM scores were compared across all available visits; however significant differences were found when changes from baseline to specific time endpoints were compared separately using a conventional univariate analysis of variance (ANCOVA) approach. Groups were compared at each treatment timepoint (V3, V4, V5, V6) and LOCF separately while adjusting for baseline (V2) score. Groups differed significantly in the change from V2 (baseline) to V4 (one month on treatment and after two weeks with NSAID dose at 50%). Significantly higher ROM for passive and active extension (0.83° ± 1.54 vs. -1.54° ± 2.43; difference, 5.2° ± 2.2, p = 0.028) were recorded for the Aquamin group (Figure 2 and Table 4) following the 50% reduction in NSAID use. No statistically significant changes were noted between the groups for active or passive flexion ROM.

### 6 MWD

**Figure 3:**
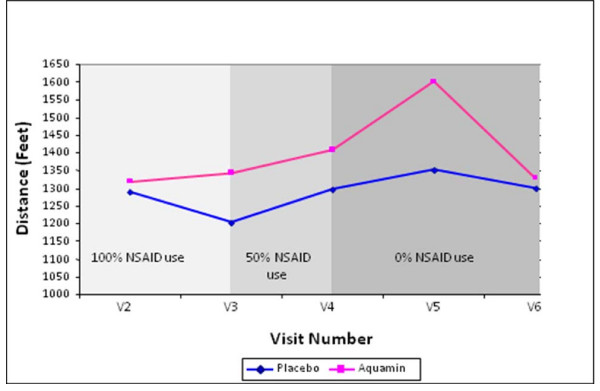
**Walking distance over time**. Change in distance (Feet) walked over 12 weeks of treatment.

No differences were found in 6 MWD when scores were compared across all visits from V2 to V6; however, analysis of each time-point separately while adjusting for the baseline (V2) score showed significant differences between the two groups in the change from V2 (baseline) to V4 (one month on treatment and after two weeks with NSAID dose at 50%). The distance covered during a 6 minute timed walk was significantly improved within the Aquamin group compared to the placebo group (150 ± 48 ft vs. 12.5 ± 31.5 ft; difference, 136 ± 57 ft, p = 0.03) (Figure 3 & Table 4) indicating a significant improvement in the Aquamin treated group when NSAID use was reduced by 50%.

### Adverse events

All treatments were well tolerated and no serious adverse events were reported in this trial. Table 5 shows the 31 adverse events (AE) that were reported with 10 AE in the Aquamin group and 21 in the placebo group. One adverse event was scored as at least possibly related to the study due to the timing of the gastrointestinal-related disturbance and the cessation of the problem with end of treatment (however this subject was in the placebo group). All AE have completely resolved.

**Table 5:** Adverse events

Item	Placebo	Aquamin	Total
Number subjects treated	14	8	22
Total number of adverse events	21	10	31
-upper respiratory events	3	2	5
-gastrointestinal events	4	1	5
-food reaction	0	1	1
-hypertension/cardiovascular	1	1	2
-musculoskeletal events	4	3	7
-neurological events	2	0	2
-increased OA pain	7	2	9

## Discussion

This preliminary study was designed to investigate the potential of a marine derived multi-mineral supplement (Aquamin) to allow for reduced NSAID usage over three months in subjects with moderate to severe OA of the knee. Early in the trial, six subjects discontinued participation because subjects described increased knee pain and did not wish to continue in the trial. These subjects were treated on average until day 45 +/- 16 days (mean +/- SD) (range 28 to 65 days) and the timing of their withdrawal coincided with the time they were stopping their NSAID use (i.e. after four weeks or 28 days on study treatment). Of interest, five of the six subjects who withdrew due to increased knee pain were on placebo and only one was receiving Aquamin. The five subjects on placebo withdrew at the following days of treatment: 28, 32, 39, 41, and 64 days and the one subject on Aquamin had the longest time on treatment (65 days) before discontinuation. This suggests that none of the subjects dropped out of the trial when they were at 50% of their NSAID dose; however, 27% (6/22) withdrew due to increasing pain after stopping their NSAID use entirely. Although not statistically significant, 36% (5/14) of subjects withdrew from the placebo group while only 13% (1/8) withdrew from the Aquamin group due to increasing pain after stopping their NSAID use. Although more research is needed, these data suggest that Aquamin treatment compared to placebo may have been helpful to keep people in the trial (with less pain in the affected knee) while they reduced their NSAID use by 50–100%.

Of interest, placebo and Aquamin treatments were significantly different in the change from V2 (baseline) to V4 (one month on treatment and after two weeks with NSAID dose at 50%) with higher adjusted mean values for ROM for passive and active extension (173° vs. 168°; p = 0.028) as well as a greater six-minute walking distance (1408 ft vs. 1295 ft; p = 0.03) for subjects on Aquamin compared to placebo. This was an improvement of 8.7% over the distance walked at baseline. Although, these distances appear to be small, our subjects with severe OA indicated the ability to walk even a little bit further was important to them. It is interesting to underline that these significant results at V4 compared to V2 were recorded not only following Aquamin treatment but also while NSAID dosage was reduced by 50%. These results suggest that Aquamin may provide some improved function in the setting of a 50% reduced but not eliminated NSAID dose.

It should be noted that the positive results did not continue once NSAID use was abolished completely. Thus, Aquamin cannot entirely replace NSAIDs as a treatment for OA. However, Aquamin may allow for a reduced need for NSAIDs which may have substantial health benefits including a reduction in many of the adverse and well documented side effects of NSAIDS. Further study is needed to verify this information and to explore the ability of Aquamin to improve walking distance and range of motion for subjects with osteoarthritis of the knee.

The limitations to this study include the short duration of treatment (12 weeks) and limited sample size; however, the extent of the daily pain endured by sufferers of OA is highlighted by the fear and reluctance of these subjects to completely eliminate NSAID usage for eight consecutive weeks. Although the differences reported are small, to the subjects with moderate to severe OA, the ability to walk and move more freely is of enormous importance. Additional studies of longer treatments in a greater number of subjects are necessary to fully explore the treatment effect of Aquamin in OA.

Aquamin is composed of multiple minerals and the 'active ingredient' for the complex is difficult to determine. A number of minerals in Aquamin may have anti-inflammatory and anti-oxidant properties which may directly and/or indirectly influence the efficacy of this unique complex. While the prominent mineral present in Aquamin is calcium (dosage = 80% calcium RDA) its role in joint health remains unclear and warrants further study. Magnesium, was given at the daily dosage providing 14% (male) to 18% (female) US RDA. This may have influenced OA symptoms by affecting the utilization of calcium or by potentially reducing the inflammation around the affected joint. Both manganese and selenium were given at the daily dosage providing up to 16% and 4% of their RDA respectively. Both of these trace minerals have been reported to reduce the appearance of osteoarthritic lesions and reduce the severity of symptoms in OA.

## Conclusion

This study suggests a potential treatment effect for Aquamin among sufferers of moderate to severe OA and a possible reduction in the need for NSAIDs to manage the symptoms of OA. These preliminary findings warrant further study.

## Competing interests

Marigot Ltd provided funding for this pilot trial and the costs to publish this work. The authors declare that they have no other competing interests. Marigot Ltd approved the protocol, reviewed the manuscript before submission for publication and can be reached at: Strand Farm, Currabinny, Carrigaline, Co. Cork, Ireland. Phone: 353-21-437-8727; Fax 353-21-437-8588. Marigot Ltd did not participate in any of the data collection reported herein.

## Authors' contributions

JLF co-authored the protocol and directed the research team at MARC during the conduct of this trial and provided critical review and revision of the manuscript. JLZ co-authored the protocol, provided critical review of the manuscript and provided medical monitoring services during the trial. MAK provided critical review of the protocol and manuscript and provided statistical services for the design, execution and analysis of the data in this trial. All authors have read and approved the final manuscript.
